# Supplementary material for: Identification and functional analysis of non-coding regulatory small RNA FenSr3 in Bacillus amyloliquefaciens LPB-18
Source: PeerJ. 2023 May 15;11:e15236. doi: 10.7717/peerj.15236 (PMC10194069; doi:10.7717/peerj.15236)
Supplement: Supplemental Information 4 [file peerj-11-15236-s004.zip › KO/CK-vs-T1_map/map05218.html]

KEGG PATHWAY: Melanoma - Reference pathway


|  |  |
| --- | --- |
| **Melanoma - Reference pathway** |  |

[
Pathway menu
| Organism menu
| Pathway entry
| Show description
| User data mapping
]

Melanoma is a form of skin cancer that has a poor prognosis and which is on the rise in Western populations. Melanoma arises from the malignant transformation of pigment-producing cells, melanocytes. The only known environmental risk factor is exposure to ultraviolet (UV) light and in people with fair skin the risk is greatly increased. Melanoma pathogenesis is also driven by genetic factors. Oncogenic NRAS mutations activate both effector pathways Raf-MEK-ERK and PI3K-Akt. The Raf-MEK-ERK pathway may also be activated via mutations in the BRAF gene. The PI3K-Akt pathway may be activated through loss or mutation of the inhibitory tumor suppressor gene PTEN. These mutations arise early during melanoma pathogenesis and are preserved throughout tumor progression. Melanoma development has been shown to be strongly associated with inactivation of the p16INK4a/cyclin dependent kinases 4 and 6/retinoblastoma protein (p16INK4a/CDK4,6/pRb) and p14ARF/human double minute 2/p53 (p14ARF/HMD2/p53) tumor suppressor pathways. MITF and TP53 are implicated in further melanoma progression.

|  |  |  |
| --- | --- | --- |
| Reference pathway | 184% 150% 122% 100% 82% 67% 55% | 图片下载 |
